# Allosteric Effects between the Antibody Constant and Variable Regions: A Study of IgA Fc Mutations on Antigen Binding

**DOI:** 10.3390/antib7020020

**Published:** 2018-06-07

**Authors:** Chinh Tran-To Su, Wai-Heng Lua, Wei-Li Ling, Samuel Ken-En Gan

**Affiliations:** 1 Bioinformatics Institute, Agency for Science, Technology and Research (A*STAR), Singapore 138671, Singapore; chinhsutranto@bii.a-star.edu.sg (C.T.-T.S.); luawh@bii.a-star.edu.sg (W.-H.L.); lingwl@bii.a-star.edu.sg (W.-L.L.); 2 p53 Laboratory, Agency for Science, Technology and Research (A*STAR), Singapore 138648, Singapore

**Keywords:** antibody, isotype IgA, Pertuzumab, allosteric, biologics, constant region, variable region

## Abstract

Therapeutic antibodies have shifted the paradigm of disease treatments from small molecules to biologics, especially in cancer therapy. Despite the increasing number of antibody candidates, much remains unknown about the antibody and how its various regions interact. Recent findings showed that the antibody constant region can govern localization effects that are useful in reducing side effects due to systemic circulation by the commonly used IgG isotypes. Given their localized mucosal effects, IgA antibodies are increasingly promising therapeutic biologics. While the antibody Fc effector cell activity has been a focus point, recent research showed that the Fc could also influence antigen binding, challenging the conventional idea of region-specific antibody functions. To investigate this, we analysed the IgA antibody constant region and its distal effects on the antigen binding regions using recombinant Pertuzumab IgA1 and IgA2 variants. We found that mutations in the C-region reduced Her2 binding experimentally, and computational structural analysis showed that allosteric communications were highly dependent on the antibody hinge, providing strong evidence that we should consider antibodies as whole proteins rather than a sum of functional regions.

## 1. Introduction

Antibodies, called the “magic bullet” by Paul Erhlich [1,2,3], have shown great promise as therapeutic agents against numerous diseases [4], with many breakthroughs documented [5,6,7,8,9,10]. One promising isotype is IgA, whose predominant mucosal activation and localization can reduce systemic circulations and the associated side effects over the reigning IgG isotypes [9,10,11].

IgA (IgA1 and IgA2) is the major immunoglobulin isotype in adaptive mucosal immunity [12,13,14,15] and is responsible for several disease pathologies such as IgA nephropathy when they polymerize or self-aggregate [16,17]. Recently, a chimeric IgG-A antibody (with engineered CHγ2–CHα3 Fc region [18]) showed greater killing of Her2+ cancer cells by higher levels of complement-dependent cytotoxicity and activations of both neutrophils and macrophages. Although the chimeric IgG-A utilized only the CHα3 domain, this example clearly showed that the Fc of antibodies could be engineered towards various effector effects.

Fc manipulations have also been used to improve antibody half-lives [19] as well as to make bispecific antibodies [8] or create “sweeping” antibodies [20]. However, the overall effects of such constant region modifications on other antibody functions such as antigen bindings are not well established. Recently, there are increasing reports [21,22,23,24,25,26] of distant effects (likely allosteric communications) between the Fc region and the antigen-binding regions, with these studies typically based on IgG antibodies.

Similar to the case of IgG [22,26], our previous work [25] demonstrated that the heavy-chain constant regions can modulate antigen binding, most obviously for IgM and IgD, and to a lesser extent for IgA and its subtypes. To further investigate these effects, we generated mutations in the IgA constant regions and measured the antigen binding experimentally alongside computational analyses of allosteric communications between the constant and variable regions of these IgA antibodies.

## 2. Materials and Methods

### 2.1. Production of Recombinant Pertuzumab IgA Antibodies

Wild-type recombinant Pertuzumab IgA1 and IgA2 were synthesized and expressed as were previously described [25]. The mutations (C266Y/H317R for IgA1 and C253Y/H304R for IgA2): a conserved cysteine and the other residue randomly picked as a control were incorporated into the IgA constant regions by site-directed mutagenesis (Agilent Technologies, Santa Clara, CA, USA, Cat no. 200521). Produced IgA antibodies were quantified by spectrophotometric means using the extinction coefficient values determined from ProtParam [27]. Gel filtration figures were generated from Unicorn 6.0 software (GE Healthcare, Marlborough, MA, USA) with lines thickened using the GIMP 2.9.4 software. Purified antibody variants were analysed on reducing 10% SDS-PAGE gels and stained using Bio-Safe Coomassie stain (Bio-Rad, Hercules, CA, USA, Cat no. 1610786). Gel band sizes were determined using GelApp [28].

### 2.2. Binding Affinity Studies

Binding kinetics (using Blitz^®^, Fortebio, Fremont, CA, USA) of the antibodies to Her2 were carried out by pre-binding of HIS-tagged Her2 (Sino Biologicals Inc., Wayne, PA, USA, Cat no. H10004-H08H) onto the Ni-NTA (NTA) biosensors (Fortebio, Fremont, CA, USA, Cat no. 18-5101) as previously described and performed [25,29] using 1× kinetic buffer.

### 2.3. Modelling Full Antibody Structures of IgA1 and IgA2

Atomistic models of the two antibody variants IgA1 and IgA2 were constructed using two scattering-solved structures PDB: 2QTJ and PDB: 1R70 as templates for the Fc region, respectively. PDB: 1S78 was used as the template for the Pertuzumab Fab region. The resulting Cα-based backbones of the Fc regions were then used to construct the full-atom backbones and side chains using PULCHRA [30] and SCWRL4 [31], respectively. A standard procedure of energy minimization (5000 steps using steepest descent followed by conjugate gradient) was performed to remove possible clashes, using AMBER 14 [32]. Mutant IgA1 and IgA2 structures were modelled with corresponding mutations C266Y/H317R and C253Y/H304R, respectively.

The energy-minimized structures of the two variants (each including the wild type and mutant) were then subjected to coarse grain simulation (using Martini force field for proteins combined with *ElNeDyn* elastic network) to sample conformational changes of the whole antibody structures. The simulations were performed with time steps (*dt*) gradually increased from 15 fs to 22 fs during the equilibrium to accommodate ion wild motions, then fixed at *dt* = 22 fs during the production stages (3 × 1 μs) with the Verlet algorithm. Periodic boundary condition was also applied to avoid the finite size effects while simulating in explicit solvent (polarized water model; hence with PME). Temperature and pressure coupling schemes were used with the velocity rescale (*V-rescale*) and the Parrinello–Rahman barostat. Our analyses used the data from the last 600 ns (×3) of the simulated trajectories that reflected stable simulations, resulting in 3 × 1000 conformations.

### 2.4. Quantification of Allosteric Effects

**Figure 1:**
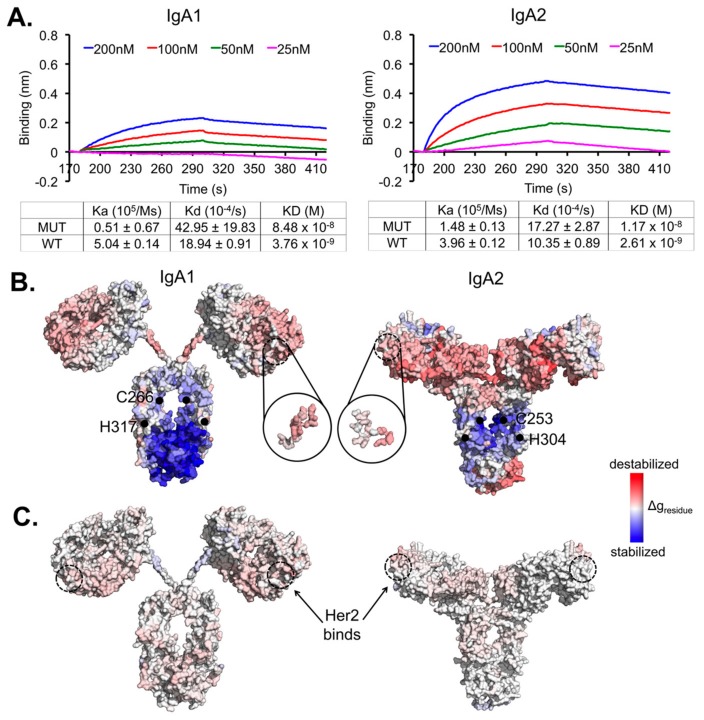
Synergistic allosteric effects by the two IgA1 and IgA2 constant region mutations on the Her2-binding variable regions. (**A**) Binding kinetics analysis of the isotype variants IgA1 and IgA2 to Her2, using the antibodies at 200 nM to 25 nM to pre-loaded Her2 on NTA biosensors. The binding kinetics was measured using Blitz^®^. All experiments were performed in triplicates independently. The binding kinetics values of the wild-type IgA1 and IgA2 shown were obtained from our previous work [25]. (**B**) Surface presentations of the quantified allosteric communications (presented by residual allosteric free energy change Δg_residue_) demonstrate destabilizing effects on the Her2 binding region caused by the mutations (black dots) in both the mutant constant region variants. (**C**) The quantified allosteric effects shown were based on the event of Her2 bindings. In (**B**,**C**), the effects were estimated using the minimized structures of both the wild-type IgA subtypes for perturbations with respect to mutating or binding events.

We first used the minimized structures of the wild-type variants IgA1 and IgA2 to quantify the allosteric effects in both the Her2-binding and mutation events (as shown in Figure 1) using the server AlloSigMA [33], which have demonstrated successful quantification of allosteric effects in various benchmarked allosteric proteins [34,35,36,37]. The allosteric communications were estimated based on the responses of each residue (via residual free energy change Δg_residue_) with respect to perturbations due to each of the events [33]. In this analysis, we simulated the mutations by initiating perturbations at the substituted positions (i.e., assigning “Up-mutation” in the AlloSigMA server to simulate larger residue substitutions). The resulting residue-wise allosteric free energies (with negative values indicating stabilizing and positive values indicating destabilizing effects) showed the quantified allosteric effect caused by the mutations. We then estimated the free energy change at the Her2 binding site (Δg_Her2site_) and other corresponding regions (Δg_region_) by averaging all Δg_residue_ values of the involving residues.

**Figure 2:**
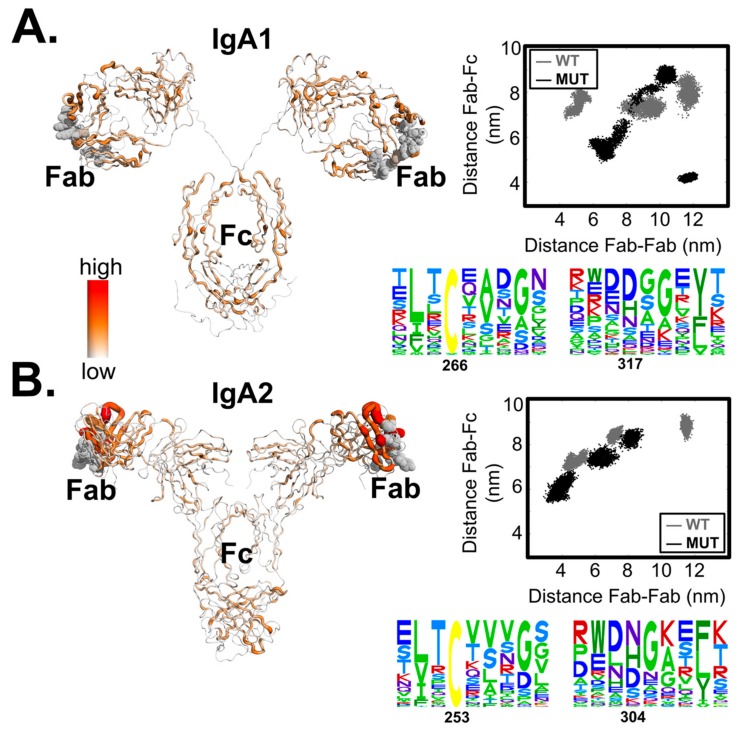
Residue couplings and hinge-dependent domain proximity of the two variants IgA1 (in **A**) and IgA2 (in **B**). The sequence alignments of the mutation regions were performed using the EVcoupling server [39] and the coupling values were mapped back to the minimized structures of the variants. Distances were estimated between the centre of mass of the Fc region and that of the Her2-binding sites. Note that in the distance distributions of the IgA2 mutant (in **B**) showed the plotted values obtained from two replicates that successfully reached equilibrium in the given time scales.

In addition, we estimated and clustered the distance ratio (i.e., distances between centre-of-mass of Fab regions versus those of both Fab and Fc as shown in Figure 2) of the wild-type structures resulting from the coarse grain simulation. Conformations nearest to the centroids were extracted and reverted to atomistic structures using Charmm36 force field and TIP3P water model, followed by short minimization and equilibrium. The structures were then used to study the spectrum of the allosteric effect driven by the domain motions.

### 2.5. Data Availability

The datasets generated and/or analysed during the current study are available upon reasonable request.

## 3. Results

Our previous work [25] suggested that the antibody heavy chain constant regions, but not light chain constant regions, influenced antigen binding beyond simple avidity effects, e.g., expected for IgM. To further investigate this phenomenon, we generated several disruptive mutations: C266Y/H317R in IgA1 and C253Y/H304R in IgA2 at the heavy chain constant region (CHα3) of our Pertuzumab IgA1 and IgA2 (i.e., intentionally substituting one of the disulphide-forming cysteine residues in the CH3 domain with the bulky residue tyrosine and another randomly selected histidine residue with similar positively charged arginine) to affect the heavy chain stability, and study the corresponding effects on the Her2-binding region.

Experimentally, we found the recombinant transient expression of both mutant variants to drop drastically (multiple folds) compared to the wild types (data not shown), implying the important role of the conserved disulphide-forming cysteine in the antibody stability [38]. A higher rate of aggregation in the isotype mutants was also observed (Figure S1). In addition, our binding kinetics measurements showed a significant decrease of the mutants by a log at 10^−8^ compared to the wild type at 10^−9^ for both IgA1 and IgA2. The major effects on IgA1 were at the association constant measurements for IgA1 and to a reduced extent, at the dissociation constant; for IgA2, the differences were less pronounced on both the association and dissociation constants (Figure 1A).

Our experimental measurements show that mutation-driven perturbations in the constant regions can affect Her2 binding, even with a few substitution mutations. This suggests clear allosteric communications between the two regions. We applied a structure-based statistical mechanical model [37] (using the AlloSigMA server [33]) to quantify these underlying allosteric effects. Results showed that the mutations caused stabilizing effects in the Fc region (Figure 1B and Table 1) that was compensated by increasing energy gain in distant regions (V-regions), thus destabilizing the Her2 binding site, which became more flexible (with Δg_Her2site_ > 0) due to contact losses between the Her2-interacting residues and their neighbours (Table 2). This destabilizing effect is more pronounced in the IgA1 mutant (Table 3 and Figure S2) than in IgA2.

We independently initiated computational perturbations at the Her2-binding site and studied the communication to the Fc in both the variants to simulate the bi-directional signal propagations between the two regions (Figure 1B,C). When comparing between the two events of mutations and Her2-binding, we noticed energy compensation to occur at the Fab regions of both IgA1 and IgA2. The IgA2-Fab domain compensated significantly more than that of IgA1 on the whole, distributing the energy compensation across the entire V-region, thus balancing the destabilization at the Her2 binding site (as $\Delta g_{Her2site}^{IgA2}$ < $\Delta g_{Her2site}^{IgA1}$) and retaining more binding to Her2. On the other hand, the energy compensation in the IgA1 Fab partially accommodated the changes at the Proline-rich hinge in terms of rigidity (Figure 1B). This might not be the case for the IgA2 short hinge, suggesting that the allosteric communication barrier was lifted with increasing hinge flexibility (Table 1 and Table 3).

Meanwhile, we hypothesized that the two mutation positions have functional selection constraints. To test this, we used the EVcoupling server [39] to investigate the residue couplings of the constant and variable regions (to demonstrate the local proximity as well as contacts in the antibody variants under the function dependent constraints). We found strong residue coupling networks forming independently within domains Fab or Fc, but weak links between the two domains (Figure 2). In the Fc domain, only the cysteine residue (C266 in IgA1 or C253 in IgA2) exhibited moderate functional dependence, which was expected for a conserved cysteine.

When additionally performing single perturbations at the individual positions, we found the allosteric signals to be elicited by the substitution of the histidine (H317 in IgA1 or H304 in IgA2) with a larger residue. Interestingly, the highly conserved disulphide-forming cysteine contributed to less destabilizing effects on the Her2 binding site (Table 3). These results indicate that the two domains Fab and Fc clearly communicate with each other, and that the histidine position might cause a bigger allosteric effect. We also found the destabilizing effect on the Her2-binding site to be a result of accumulative signalling, facilitated via the hinge. Therefore, the effect caused by the Fc mutations on the Her2 binding ability of the Fab domain (Figure 1A) is modulated by hinge flexibility.

The IgA1 isotype contains a longer hinge (connecting CH1 and the rest of the Fc region), the flexibility of which amplified with the mutation events (with $\Delta g_{hinge}^{IgA1}$ = 0.21 ± 0.20 whereas $\Delta g_{hinge}^{IgA2}$ = 0.08 ± 0.05). Scattering experiments on the wild-type IgA1 isotype (PDB: 2QTJ) showed that a rigid flanking hinge that separates Fab and Fc in an extended IgA1 conformation in solution is favoured. Our dynamics simulations of the wild-type IgA1 structure also showed that the two regions remained in constant proximity to each other. Nonetheless, diverse domain fluctuations between the two regions were observed in the conformational sampling of the IgA1 mutant (Figure 2A, top right), implying that mutations caused structural interferences and hence the hinge flexibility. On the other hand, the shorter IgA2 hinge did not allow for wild domain motions (Figure 2B, top right). Since the Her2-binding ability is mostly abolished in the IgA1 mutant but is retained in the IgA2 mutant, there is evidence that hinge flexibility modulates the propagation of the allosteric signals between the two distant domains.

## 4. Discussion

We set out to investigate the mechanism of the allosteric effects that the antibody constant regions elicited on the antigen binding as suggested in our previous work [25]. Working on our IgA1 and IgA2 models, we sought to study such effects in greater details by analysing the effects of Her2 binding when two mutations were introduced, at a conserved cysteine (C266/C253) and a random selected control histidine (H317/H304). While we acknowledge that the starting structure dependency remains a challenge in our approach, we have agreement between the computational and experimental results, where the computational observations could provide us with insight into the allostery phenomenon between the variable and constant regions.

Experimentally, we found that the double mutants were produced at a significantly reduced rate in our transient expressions, and that there were compromises in terms of binding ability to Her2, albeit at different magnitudes, despite unmodified variable regions. This further demonstrated that the constant region had clear significant effects on antigen binding, agreeing with other such studies on IgGs [21,22,24,40], in the IgA context. To understand the mechanism underlying our experimental observations, we showed that the allosteric signalling propagated bi-directionally between the two distant regions, from Her2-binding region to the Fc region and vice versa, on the IgA isotypes. In fact, the domain-linking hinge mediated such communication signalling, demonstrating that the hinge flexibility modulated the level of energy compensations at the distal regions. 

We postulated that the energy compensation at the Fabs of the IgAs variants to accommodate the flexibility changes upon balance at the Her2-binding site (Table 1 and Table 3) to maintain the Her2-binding ability. As a result, our findings are in partial agreement with several previous studies [41,42,43] that the antigen-binding region requires a certain level of rigidity. On the contrary, other studies [44,45] suggested no significant conformational differences found in these regions, perhaps due to the different antigen binding contacts. It should also be noted that many of these previous studies were performed on antibody fragments, e.g., Fab or Fv, or on IgG, and not on the whole IgA. Thus, together with our other findings [25,26,46,47] also involving other proteins, we have shown the need to examine proteins as a whole for comprehensive holistic investigations, especially on allostery. In the context of the whole antibody, we found that the hinge rigidity and the allosteric communications between the distant constant and variable regions both contribute to antigen binding.

Our results here are also consistent with other previous studies [21,23] that showed induced conformational changes at the Fc due to the binding of antigen, hence consequently regulating the Fc receptor binding. Since there might also be other residues in the Fc region that could allosterically affect the antigen-binding site, we believe there would be a network of allosteric residues that drive the allosteric signalling between the regions. Our future work will thereby explore hotspots or mutation boundaries that can improve antigen binding by taking the advantages of allosteric communications from constant regions towards a rational and targeted approach for Fc engineering.

In conclusion, using both experimental and computational approaches, we were able to show that the constant region is important in the ability of the variable regions in binding antigens. We showed this in the less studied IgA, and that such effects were computationally found to be mediated by the hinge region of the antibody. This further illustrates the need to consider antibodies as a whole, rather than merely a sum of its regions.

## Figures and Tables

**Table 1:** Accumulative allosteric effect on various regions (represented by Δg_region_) using the minimized structures of IgA1 and IgA2, when mutated or when bound to Her2.

	Δg_region_ (kcal/mol)			
	Mutating Event		Her2-Binding Event	
Region	IgA1	IgA2	IgA1	IgA2
Her2 binding site	0.18	0.12	0.06	−0.05
Fab	0.15	0.38	0.08	0.02
Fc (CH2–CH3)	−0.46	−0.08	0.03	−0.007
Hinge	0.05	0.04	−0.02	0.03

**Table 2:** Percentages * of native contacts (%) in the minimized structures of IgA1 and IgA2 mutants, showing the contact loss as compared to the wild type.

	All Heavy Atoms/Cα Atoms	
	IgA1	IgA2
Wild type	100/100	100/100
Mutant (replicate 1)	69.7/58.3	57.1/61.8
Mutant (replicate 2)	66.5/56.7	55.7/61.8
Mutant (replicate 3)	66.5/56.7	54.7/61.8

* Percentage = (number of native contacts in mutant)/(number of native contacts in wild type).

**Table 3:** Allosteric free energy (Δg_region_) estimated in mutation events (accumulative or single mutations) using different conformations of the wild-type variants IgA1 and IgA2.

	Δg_region_ (kcal/mol) in the Event of Mutations					
	IgA1 (C266/H317)	IgA2 (C253/H304)	IgA1 (C266)	IgA2 (C253)	IgA1 (H317)	IgA2 (H304)
Her2 site	0.38 ± 0.32	0.12 ± 0.06	0.18 ± 0.16	0.06 ± 0.07	0.28 ± 0.23	0.09 ± 0.05
Fab	0.62 ± 0.54	0.31 ± 0.12	0.28 ± 0.26	0.23 ± 0.09	0.5 ± 0.36	0.13 ± 0.08
Fc (CH2–CH3)	−0.52 ± 0.05	−0.16 ± 0.27	−0.4 ± 0.07	−0.06 ± 0.29	−0.25 ± 0.09	−0.1 ± 0.03
Hinge	0.21 ± 0.2	0.08 ± 0.05	0.08 ± 0.09	−0.003 ± 0.032	0.20 ± 0.17	0.07 ± 0.04

## Supplementary Materials

The following are available online at http://www.mdpi.com/2073-4468/7/2/20/s1, Figure S1: Pertuzumab isotype/subtype biophysical analysis. Figure S2: Number of contacts within 6 Å (0.6 nm) of the Her2-interacting residues of the two variants IgA1 and IgA2.
