# Supplementary material for: Allosteric Effects between the Antibody Constant and Variable Regions: A Study of IgA Fc Mutations on Antigen Binding
Source: Antibodies (Basel). 2018 Jun 7;7(2):20. doi: 10.3390/antib7020020 (PMC6698812; doi:10.3390/antib7020020)
Supplement: Supplementary file 1 [file antibodies-07-00020-s001.pdf]

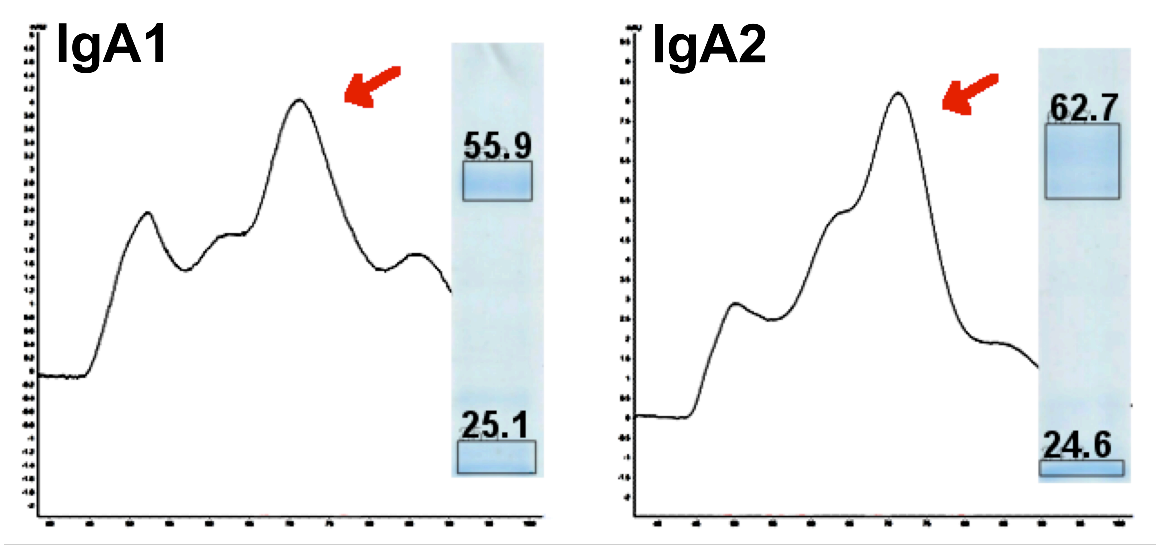

Figure S1: Pertuzumab isotype/subtype biophysical analysis. Gel Filtration profiles of the Pertuzumab variants following affinity purification using Superdex size exclusion column on AKTA Pure. X-Axis: 40-100 minute time scale. Y-axis: mAU as determined by UV detection. Red arrows depict the selected peak fractions of the antibodies used for subsequent analyses. SDS-PAGE analyses of concentrated antibodies (reduced) are shown in the smaller inserts with corresponding band sizes for the light and heavy chain. Gel band sizes were determined using GelApp [1].

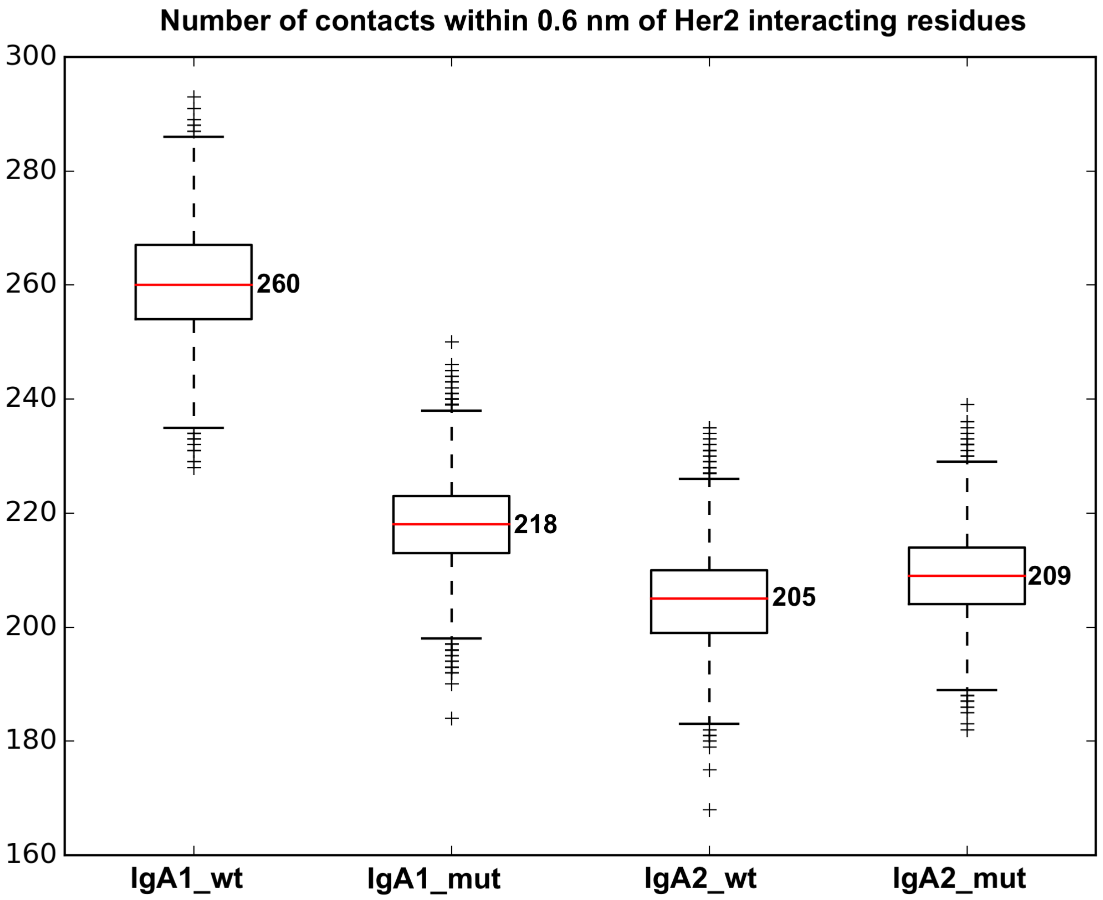

Figure S2: Number of contacts within 6Å (0.6 nm) of the Her2-interacting residues of the two variants IgA1 and IgA2. The contacts were estimated using the last 600ns trajectories of the coarse grain simulation.

Reference:

1. Sim, J.Z.; Nguyen, P.V.; Lee, H.K.; Gan, S. GelApp: mobile gel electrophoresis analyser. *Nat. Methods Application Notes* **2015**.
